# The “better data, better planning” census: a cross-sectional, multi-centre study investigating the factors influencing patient attendance at the emergency department in Ireland

**DOI:** 10.1186/s12913-022-07841-6

**Published:** 2022-04-09

**Authors:** Niamh M. Cummins, Louise A. Barry, Carrie Garavan, Collette Devlin, Gillian Corey, Fergal Cummins, Damien Ryan, Sinead Cronin, Emma Wallace, Gerard McCarthy, Rose Galvin

**Affiliations:** 1 School of Medicine, Faculty of Education and Health Sciences, University of Limerick, Limerick, Ireland; 2 SLÁINTE Research and Education Alliance in General Practice, Primary Healthcare and Public Health, Faculty of Education and Health Sciences, University of Limerick, Limerick, Ireland; 3 Ageing Research Centre, Health Research Institute, University of Limerick, Limerick, Ireland; 4 School of Allied Health, Faculty of Education and Health Sciences, University of Limerick, Limerick, Ireland; 5 Department of Nursing and Midwifery, Faculty of Education and Health Sciences, University of Limerick, Limerick, Ireland; 6 Emergency Department, ALERT Limerick EM Education Research Training, University Hospital Limerick, Limerick, Ireland; 7 Health Research Board Centre for Primary Care Research, Royal College of Surgeons in Ireland, Dublin, Ireland; 8 Emergency Department, Cork University Hospital, Cork, Ireland

**Keywords:** Emergency Department, Crowding, Access to Care, Alternative Care Pathways, COVID-19

## Abstract

**Background:**

Internationally Emergency Department (ED) crowding is a significant health services delivery issue posing a major risk to population health. ED crowding affects both the quality and access of health services and is associated with poorer patient outcomes and increased mortality rates. In Ireland the practising of “Corridor Medicine” and “Trolley Crises” have become prevalent. The objectives of this study are to describe the demographic and clinical profile of patients attending regional EDs and to investigate the factors influencing ED utilisation in Ireland.

**Methods:**

This was a multi-centre, cross-sectional study and recruitment occurred at a selection of urban and rural EDs (*n* = 5) in Ireland throughout 2020. At each site all adults presenting over a 24 h census period were eligible for inclusion. Clinical data were collected via electronic records and a questionnaire provided information on demographics, healthcare utilisation, service awareness and factors influencing the decision to attend the ED.

**Results:**

Demographics differed significantly between ED sites in terms of age (*p* ≤ 0.05), socioeconomic status (*p* ≤ 0.001), and proximity of health services (*p* ≤ 0.001). Prior to ED attendance 64% of participants accessed community health services. Most participants (70%) believed the ED was the “best place” for emergency care or attended due to lack of awareness of other services (30%). Musculoskeletal injuries were the most common reason for presentation to the ED in this study (24%) and almost a third of patients (31%) reported presenting to the ED for an x-ray or scan.

**Conclusions:**

This study has identified regional and socioeconomic differences in the drivers of ED presentations and factors influencing ED attendance in Ireland from the patient perspective. Improved awareness of, and provision of alternative care pathways could potentially decrease ED attendances, which would be important in the context of reducing ED crowding during the COVID-19 pandemic. New strategies for integration of acute care in the community must acknowledge and plan for these issues as a universal approach is unlikely to be implemented successfully due to regional factors.

**Supplementary Information:**

The online version contains supplementary material available at 10.1186/s12913-022-07841-6.

## Background

A key target of the United Nations (UN) Sustainable Development Goals in Population Health is to improve access to essential health services and to strengthen capacity for management of national and global health risks [1]. Internationally Emergency Department (ED) crowding is a significant health services delivery issue posing a major risk to population health. ED crowding affects both the quality and access of health services and is associated with poorer patient outcomes and increased mortality rates [2].

ED crowding is referred to colloquially as “a trolley crisis”, “boarding”, “warehousing” and “corridor medicine”. Ambulance “ramping” is also a function of ED crowding with delays in patient offloads having a knock-on effect on service delivery in the prehospital setting [3]. The causes of ED crowding are complex and multifactorial, relating to input, throughout and output factors. Input factors refer to the demand for ED services, throughput factors relate to the processes of evaluation and treatment within the ED, and output factors are associated with ED disposition [4, 5].

In Ireland input factors are driven by an aging population [6] with an increasing prevalence of chronic diseases, underdevelopment of community and primary care [7], reconfiguration of acute hospital care to a more centralised model [8] and utilisation of the ED for non-urgent attendances [9]. Access to out-of-hours care in Ireland is not standardised and there is geographic variation in resourcing and utilisation of out-of-hours care across General Practitioner (GP) clinics, Ambulance Services, EDs and Local Injury Units [10].

With regard to throughput, staffing is a factor of concern, in Ireland the number of EM consultants is 2 per 100,000 population compared to 4.1 in England and 6.4 in Australia—jurisdictions where service delivery and medical training models are comparable to the Irish setting [11]. Understaffing and overcrowding contribute to poor morale and are detrimental to staff wellbeing with one recent Irish study reporting that that 78% of nurses and 70% of doctors in the ED met the criteria for burnout [12].

Arguably the most significant contributors to ED crowding in Ireland are output factors relating to hospital bed capacity which create a discharge bottleneck within the ED (access block). Average occupancy of acute beds in Ireland stands at 95% throughout the year which is one of the highest in the OECD (Average 77%) and represents a 10% increase since the year 2000 [13]. Incidentally the number of emergency admissions has increased by approximately 30% in Ireland over the last ten years, at a rate of almost 1000 per annum from 32,000 in 2005 to 41,500 in 2016 [9].

In a Priority Setting Partnership with the James Lind Alliance, the Royal College of Emergency Medicine (RCEM) recently identified methods of reducing the risks of ED crowding as the most important research priority in the field of Emergency Medicine [14]. RCEM have also highlighted the need to collect highly accurate data in order to acquire meaningful information that facilitates urgent care planning in the future [15, 16]. In Ireland, the Sláintecare Action Plan aims to improve population health by delivering the “right care, in the right place, at the right time, by the right team” [17]. The objective of this strategy is to shift the majority of care from the acute to the community setting. It is imperative that the development of new models of integrated care to address the risks of ED crowding will be data-driven and evidence-based in order to ensure successful implementation of Sláintecare.

A combination of physiological, psychological and social factors impact the decisions of patients to present to the ED. Previous studies indicate that ease of access, patient self-assessment of illness severity, and confidence in the quality of ED care are key drivers for presentation [18]. Therefore, ED crowding is one of the most complex challenges faced in health services research. Internationally many solutions have been trialled, with variable success, frequently because there is a mismatch between the identified causes of crowding and the strategies implemented to resolve the issue [19]. In the UK, high-quality data from sentinel sites has been collected to inform and guide the planning of future emergency care services nationally [15]. Investigating the demographics of patients utilising the ED and their specific reasons for attendance from the patients’ perspective is key to understanding and ultimately tackling the overall issue of ED crowding in Ireland.

## Methods

### Aim

The aims of the “Better Data, Better Planning” (BDBP) study are to describe the demographic and clinical profile of patients attending regional EDs and to investigate the factors influencing ED utilisation in Ireland.

### Setting

In Ireland the Health Service Executive (HSE) is the national publicly funded organisation responsible for the provision of health and social services. The HSE employs all health and social care professionals apart from GPs who work as independent contractors. A wider network of other primary care and allied health professionals (AHP) such as public health nurses (PHN), physiotherapists, occupational therapists, speech and language therapists, community pharmacists, dieticians, community welfare officers, dentists, chiropodists and psychologists also provide services for the population of hospital group as Primary Care Teams (PCT) [20]. The Irish public hospital system is financed by a combination of public and private spending. Patients generally fall under three categories – those with a General Medical Services (GMS) card, those with private insurance and patients with no medical cover. The “Medical Card” allows the use of most health services free of charge for patients meeting eligibility criteria based on income thresholds and burden of illness. This includes visits to a General Practitioner (GP), all inpatient and outpatient services in public hospitals and use of the ED. Without a Medical Card attendance at the ED costs €100 [21]) although this fee is waived with a referral letter from a GP. Ireland is the only western European country without universal coverage for primary care [22] and an out-of-pocket payment to visit a GP costs from €45–65 [23].

**Fig. 1:**
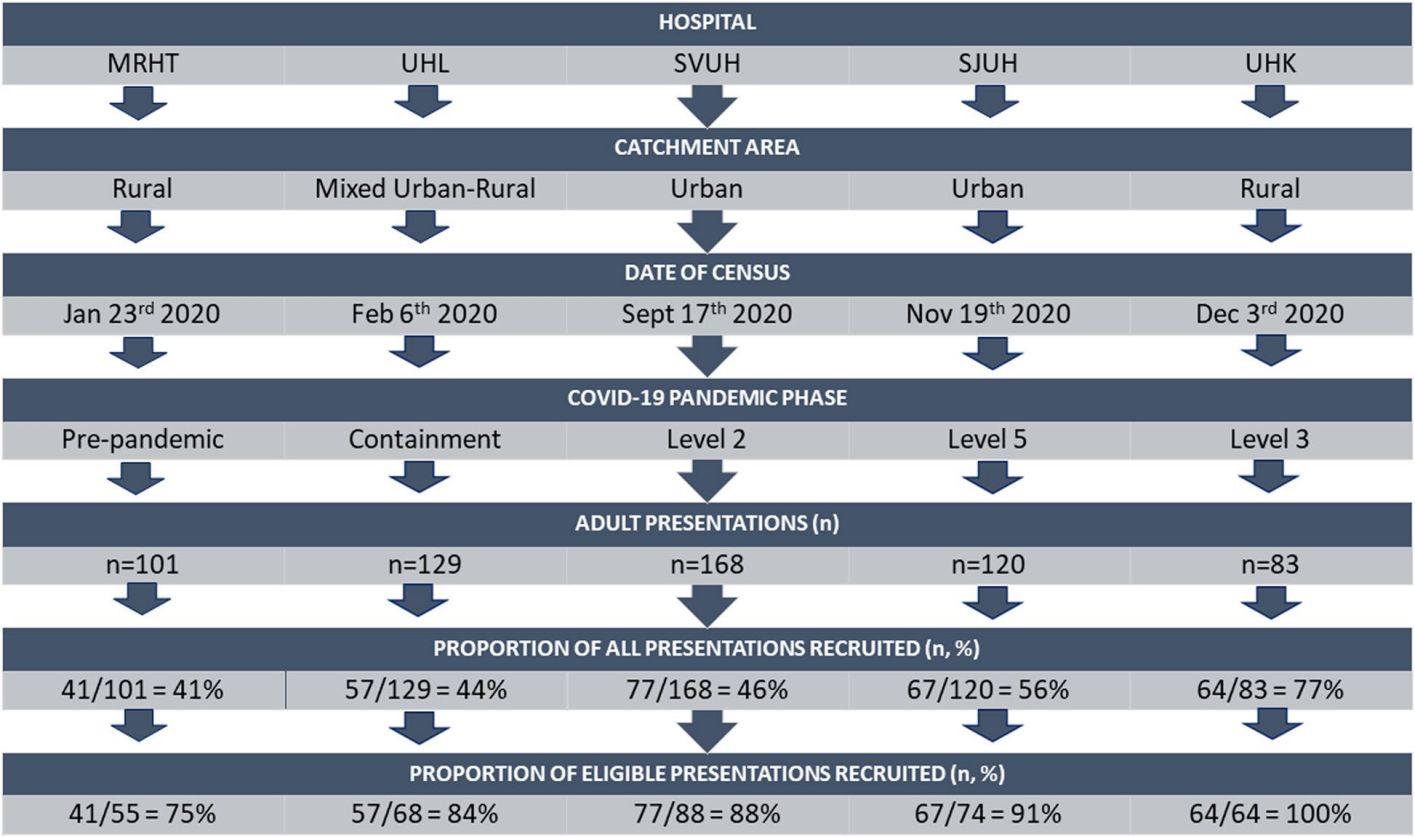
Flow Chart of the Study Population in the BDBP Study (*n* = 306). ^a^COVID-19 Pandemic Phases: Containment – Additional Public Health restrictions not yet applied. Level 2 – inter-county travel was permitted, most retail and services operating normally. Level 5 (Lockdown) – Stay at home, non-essential retail and services only. Level 3 – Local travel permitted, retail and services open with protective measures [25]

The hospitals in Ireland are organised into seven Hospital Groups; Ireland East Hospital Group, Royal College of Surgeons in Ireland (RCSI) Hospital Group, Dublin Midlands Hospital Group, University of Limerick Hospitals, South/Southwest Hospital Group, Saolta Hospital Group and Children's Health Ireland (CHI). The services delivered at these hospitals include inpatient scheduled care, unscheduled/emergency care, maternity services, outpatient and diagnostic services [24]. The setting for the BDBP study was regional EDs of selected academic teaching hospitals in urban and rural locations across Ireland (Fig. 1). Selection of hospital sites was based on a combination of geographical location, urban/rural case mix and socio-economic factors. The study includes representatives from five of the seven Hospital Groups, Midlands Regional Hospital Tullamore (MRHT); Dublin Midlands Hospital Group, University Hospital Limerick (UHL); University of Limerick Hospitals, St. Vincents University Hospital (SVUH); Ireland East Hospital Group, St. James University Hospital (SJUH), Dublin Midlands Hospital Group and University Hospital Kerry (UHK); South/Southwest Hospital Group. As the inclusion criteria specified adult participants only, the CHI hospital group was not eligible for inclusion in the BDBP Study.

### Design

This was a multi-centre, cross-sectional study profiling demographic and clinical characteristics of Irish ED attendees and investigating the factors influencing ED attendance from the patients’ perspective. This is the first report from the larger “Better Data, Better Planning” (BDBP) project investigating potentially avoidable ED attendances nationally. A pilot study was conducted in December 2019 to refine the study design and recruitment protocol, followed by trial commencement in January 2020. On 11th March, 2020 the World Health Organisation declared the COVID-19 outbreak as a pandemic [26] “Lockdown 1” commenced in Ireland on 27th March 2020 and concluded on 18th May 2020 with a phased reopening. Therefore the BDBP Census began prior to “Lockdown 1” in Ireland and the COVID-19 pandemic delayed data collection on some regional sites, which is a confounding variable in this study.

Data were collected at each site over separate 24-h periods during the course of a year to account for diurnal and seasonal variation in attendance patterns. The sampling frame for each census was Thursday at 12 pm—Friday at 12 pm. Data collection occurred at five sites throughout 2020 (Fig. 1.); January (MRHT), February (UHL) September (SVUH), November (SJUH) and December (UHK).

### Participants and procedure

All adult patients attending each ED over the sampling frame were potentially eligible for recruitment. The Manchester Triage System (MTS) is used in most Irish and UK EDs to assess the degree of severity of cases, based on presenting signs and symptoms. It assigns an order of clinical priority by allocating patients to one of five urgency categories (Immediate, Very Urgent, Urgent, Standard and Non-urgent) which determine safe waiting times in the ED [27]. The following inclusion criteria were applied A) Adult aged ≥ 18 years B) Medically stable MTS categories 2–5 C) Patient has capacity and willingness to provide informed consent. Exclusion criteria include; A) Scheduled admissions to the ED B) Mental Health presentations C) Patients with altered capacity due to drug or alcohol intoxication D) Inability to communicate sufficiently in English to participate. Patients who were COVID-19 coronavirus positive or suspected of having the viral infection were also recruited. At each site, local infection control policies and protocols were adhered to by the research team. All patients meeting the criteria, were invited to participate and served as the study denominator, with the final sample size dependant on the number of patients consenting to participate, who had sufficient time to complete the questionnaire during their ED stay. Depending on potential participants’ presenting complaint, referral route and MTS, the Research Nurses (RNs) determined when and where recruitment and informed consent could be attained within the ED (Minors, Majors, Resus). Permission was granted at each site to access electronic systems which allowed the RNs to further assess ED attendees for suitability and track their progress and allocation within the ED. In consultation with the wider Multidisciplinary Team, the RNs determined when the participant could be recruited without impacting on any treatment or diagnostics they were receiving. Patients who were initially deemed unable to participate due to e.g. pain, nausea or distress were re-assessed once they had received treatment.

Primarily, the Triage Nurses, ED Staff Nurses, Administrative Staff and Clinical Nurse Managers acted as the Study Gatekeepers and written informed consent was obtained by the RNs. Participants completed a self-report questionnaire and provided consent for access to medical charts including; demographics, public/private health insurance, socioeconomic status, presenting complaint, triage category, Length of Stay (LOS) and disposition. The questionnaire was developed by a multidisciplinary team of EM clinicians in collaboration with the researchers, it was externally reviewed, piloted internally and questions were refined based on feedback prior to full implementation. The response rate (i.e. proportion of eligible adults recruited by ED site) ranged from 75–100% and did increase chronologically from site to site (Fig. 1) which may have been a function of learning from past experience and the increasing expertise of the team of research nurses as the study progressed.

The questionnaire design incorporated open-ended questions, rating scales and multiple-choice questions. For multiple-choice questions, all responses were included in the analysis and % respondents was reported. The questionnaire explored the following categories; demographics, healthcare utilisation, service awareness and factors influencing decision to attend the ED. Demographic variables included marital status, living arrangements, education level and occupational status. Socioeconomic status was recorded by electoral division as these are the smallest legally defined administrative areas in the State for which Small Area Population Statistics (SAPS) are published from the Census. There are a total of 3,440 electoral divisions in the state, with an average population of 1,447 and average area of 20.4 square kilometres [28]. Proximity to health services (i.e. distance to GP and ED in kilometres from home address) was self-reported by participants.

Data was collected on the duration of presenting complaint and community services accessed prior to attendance. Utilisation of healthcare services in the past year was documented including hospital services (out-patient appointment, ED, hospital admission) and community services (GP, PHN and other AHP). Awareness of alternative services for emergency care including Injury Units and out-of-hours GP (OOH-GP) were also recorded. The questionnaire explored reasons for ED attendance, including self-assessment of pain and level of concern regarding presenting complaint, on a numeric rating scale [1–10].

### Patient and public involvement

Direct patient and public consultation was not undertaken, however this research was informed by previous studies detailing service user experiences of emergency care in Ireland. [8, 29].

### Data analysis

Data were entered into Excel (Microsoft, San Diego, CA), coded for analysis and analysed in SPSS (IBM SPSS Statistics Version 26, Armonk, NY). Variables were tested for normality using the Kolmogorov–Smirnov test. Categorical data are presented as frequencies and percentages and the chi-square test was used to examine relationships between variables. Continuous variables are presented as mean (standard deviation; SD) or median (Interquartile Range; IQR), depending on distribution. To compare groups, ANOVA or the Kruskall-Wallis test was used, followed by post hoc Mann–Whitney U tests. *P* < 0.05 was considered statistically significant.

## Results

### Study population and demographics

Patients meeting the inclusion criteria comprised the study denominator (Fig. 1) and the proportion of eligible participants enrolled across all sites was 306/349 (88%). Median age for the total cohort was 52y (IQR 35-69y) and the age range was 18-100y. With regard to gender, females comprised 50% and males comprised 50% of participants. The majority of participants were in a relationship (*n* = 180, 60%) and in terms of residential status most participants lived with a partner (*n* = 153, 51%). The educational level most frequently obtained was secondary level (*n* = 1131, 44%) and occupational status was recorded as employee by 39% of participants (*n* = 119). Socioeconomic status (based on electoral district) was recorded as below average or disadvantaged for 51% of the study population (*n* = 144). Most participants did not have private health insurance and accessed the ED as public patients (*n* = 143, 58%). The majority of participants (*n* = 167, 58%) lived within 5 km of their General Practitioner (GP) and within 10 km of the ED (*n* = 114, 38%). However, 8% of patients lived ≥ 15 km from their GP and 9% of patients lived ≥ 50 km from their nearest ED. The most frequently used mode of transport to the ED was by private car (*n* = 202, 67%). Full details of demographics categorised by group are provided in Table 1.

**Table 1:** Sociodemographic characteristics of study participants across BDBP hospital sites (*n* = 306)

Category	Variable	TOTAL *n* = 306	MRHT (Pre-pandemic) *n* = 41	UHL (Containment) *n* = 57	SVUH (Level 2) *n* = 77	SJUH (Level 5) *n* = 67	UHK (Level 3) *n* = 64	*P* value
**Gender**	Female	50%	59%	42%	53%	48%	50%	0.545
	Male	50%	42%	58%	47%	52%	50%	
**Age**	Median, IQR	52, 35–69	61, 49–74	52, 32–67	55, 37–72	47, 30–69	45, 34–65	0.05
	Range	18–100	24–91	18–91	18–92	19–88	19–100	
Age Category	18-39y	34%	20%	37%	29%	42%	39%	0.061
	40-64y	35%	37%	37%	36%	31%	36%	
	65y +	31%	44%	26%	35%	27%	25%	
**Civil Status**	Partner/Married	60%	64%	55%	68%	52%	58%	0.216
	Separated/Divorced	7%	5%	7%	3%	13%	6%	
	Widowed	8%	13%	4%	9%	10%	6%	
	Single	25%	18%	34%	20%	24%	30%	
**Residential Status**	Partner	51%	64%	46%	58%	46%	42%	0.372
	Family	25%	21%	25%	22%	30%	25%	
	Lives Alone	17%	13%	16%	12%	19%	25%	
	Other e.g. co-share	7%	1%	13%	8%	5%	8%	
**Principal Status**	Employee	39%	30%	42%	40%	40%	41%	0.197
	Self-employed	8%	5%	7%	12%	9%	6%	
	Family Carer	8%	18%	9%	4%	3%	11%	
	Retired	31%	43%	25%	36%	27%	30%	
	Unemployed	9%	3%	12%	4%	16%	8%	
	Student	5%	3%	5%	5%	5%	5%	
**Education**	No formal education	2%	0%	2%	1%	0%	5%	0.121
	Primary	,11%	15%	13%	12%	9%	8%	
	Secondary	44%	53%	48%	32%	50%	41%	
	Technical/Vocational	16%	23%	13%	15%	14%	19%	
	Third Level	28%	10%	25%	40%	27%	28%	
**Socioeconomic Status**	Affluent	16%	0%	0%	43%	17%	0%	0.001
	Above Average	34%	29%	45%	39%	26%	27%	
	Below Average	41%	72%	32%	18%	35%	73%	
	Disadvantaged	9%	0%	21%	0%	22%	0%	
	Very Disadvantaged	1%	0%	2%	0%	0%	0%	
**Healthcare Coverage**	Public—No Cover	2%	0%	0%	3%	4%	0%	0.05
	Public—Medical Card	56%	56%	61%	36%	61%	67%	
	Private Insurance	43%	44%	39%	61%	35%	33%	
**Distance to GP**	Median (IQR)	3, 1–7	5, 2–10	3, 1–7	2, 1–6	2, 1–5	5, 3–7	0.01
	Range	0–100	0–40	1–30	1–100	1–20	1–42	
	< 5 km	58%	46%	61%	68%	66%	42%	
	< 10 km	25%	27%	18%	17%	21%	43%	
	< 15 km	10%	20%	14%	4%	8%	7%	
	≥ 15 km	8%	7%	7%	11%	5%	8%	
**Distance to ED**	Median (IQR)	15, 5–30	26, 11–40	21, 5–31	10, 5–30	7, 3–15	25,16–32	0.001
	Range	1–160	2–64	1–70	1–160	1–70	1–70	
	< 10 km	38%	15%	33%	44%	64%	21%	
	< 25 km	26%	32%	21%	29%	27%	24%	
	< 50 km	27%	44%	35%	12%	5%	52%	
	≥ 50 km	9%	10%	11%	16%	5%	3%	
**Mode of Transport to ED**	Ambulance	19%	5%	21%	25%	17%	21%	0.001
	Private Car	67%	93%	65%	66%	48%	71%	
	Public Transport	11%	2%	9%	7%	32%	3%	
	Walk	3%	0%	5%	3%	3%	5%	

^a^Interquartile Range (IQR) 25^th^- 75^th^ percentile. *P*-value from Chi^2^-tests for categorical variables and from Kruskal–Wallis test for continuous variables

The demographic profile of patients across the full cohort differed significantly between ED sites in terms of age (p ≤ 0.05), socioeconomic status (*p* ≤ 0.001), healthcare coverage (*p* ≤ 0.05), mode of transport to the ED (*p* ≤ 0.001) and proximity of health services; distance to GP (*p* ≤ 0.01) and ED (*p* ≤ 0.001). With regard to age differences, median patient age in MRHT was 61y compared to 47y in SJUH (*p* ≤ 0.01) and 45y in UHK (*p* ≤ 0.01). Adults aged 18-39y comprised 20% of cases in MRHT in comparison to 42% in SJUH. In line with this, Older Adults (age ≥ 65y) comprised 44% of cases in MRHT compared to 27% in SJU. In terms of socioeconomic status, SVUH differed significantly from all other centres with 39% of patients attending SVUH being categorised as above average and 43% classified as affluent (*p* ≤ 0.001). None of the patients presenting at SVUH, MRHT or UHK were categorised as disadvantaged or very disadvantaged compared to 23% of presentations in UHL and 22% in SJUH. In line with this 61% of patients presenting to the ED at SVUH held private insurance compared to 33% in UHK and 35% in SJUH. Medical card holders comprised 67% of patients in UHK compared to 36% in SVUH.

Mode of transport to the ED was influenced by location of the ED with lower rates of public transport availability in rural regions (MRHT 2% vs. SJUH 32%). Ambulance utilisation ranged from 5% in MRHT to 25% in SVUH. Across all sites, patients living > 50 km from the ED were more likely to be transported by Ambulance (*p* < 0.05). Proximity to health services, by geographical location was poorer in rural regions. In terms of travel distance to the ED, in the UHK cohort 55% of patients live > 25 km from the ED, and 54% in MRHT, compared to 10% of patients in SJUH.

### Health service utilisation

Utilisation of health services over the previous year was recorded (Table 2) and hospital admission(s) were reported by 35% of the population. ED attendances were also common (51%) and multiple visits (range 2–7) were recorded by 27% of participants in the full cohort, including 5% of participants characterised as frequent users (> 4 visits/year). Significant differences were observed across sites with regard to attendances at the OPD (*p* ≥ 0.005) and utilisation of GP services (*p* ≥ 0.015). A frequency of ≥ 7 GP visits was reported by 29% of patients in MRHT. On average 18% of participants were visited by a Public Health Nurse and hospital day cases ranged from 12–24% across ED sites. A significant difference was observed also between hospitals with regard to utilisation of Pharmacy Services (Table S1).

**Table 2:** Health service utilisation in the last 12 months for participants in the BDBP Study (*n* = 306)

Health Service	Freq	TOTAL *n* = 306	MRHT (Pre-pandemic) *n* = 41	UHL (Containment) *n* = 57	SVUH (Level 2) *n* = 77	SJUH (Level )5 *n* = 67	UHK (Level 3) *n* = 64	*P* value
**Hospital Admission**	0	65%	71%	67%	64%	65%	61%	0.914
	1	21%	12%	25%	24%	18%	22%	
	2–3	11%	12%	5%	7%	17%	16%	
	4–6	2%	5%	0%	3%	0%	2%	
	7 +	1%	0%	4%	3%	0%	0%	
**Out-Patient Department**	0	59%	46%	51%	58%	61%	72%	0.05
	1	17%	12%	18%	23%	17%	13%	
	2–3	19%	24%	28%	16%	15%	13%	
	4–6	3%	15%	4%	0%	3%	0%	
	7 +	3%	2%	0%	3%	5%	3%	
**Emergency Department**	0	49%	66%	46%	51%	47%	41%	0.198
	1	24%	12%	30%	25%	26%	23%	
	2–3	22%	15%	21%	21%	24%	28%	
	4–6	3%	5%	2%	3%	3%	3%	
	7 +	2%	2%	2%	0%	0%	5%	
**Injury Unit**	0	92%	100%	88%	90%	91%	94%	0.317
	1	6%	0%	9%	7%	6%	6%	
	2–3	2%	0%	4%	3%	2%	0%	
	4–6	0%	0%	0%	0%	0%	0%	
	7 +	< 1%	0%	0%	0%	2%	0%	
**Out-of-Hours (OOH) GP**	0	74%	78%	75%	77%	72%	69%	0.670
	1	14%	10%	14%	14%	19%	14%	
	2–3	10%	10%	11%	9%	9%	14%	
	4–6	1%	3%	0%	0%	0%	3%	
	7 +	0%	0%	0%	0%	0%	0%	
**General Practitioner (GP)**	0	10%	7%	7%	11%	18%	6%	0.005
	1	19%	12%	14%	28%	15%	22%	
	2–3	37%	27%	46%	30%	49%	30%	
	4–6	17%	24%	14%	18%	11%	19%	
	7 +	17%	29%	19%	13	8%	23%	
**Public Health Nurse**	0	83%	83%	84%	82%	82%	86%	0.977
	1	6%	2, 5%	1, 2%	4, 5%	7, 11%	5, 8%	
	2–3	5%	1, 2%	5, 9%	4, 5%	5, 8%	1, 2%	
	4–6	2%	2, 5%	2, 4%	3, 4%	0, 0%	0, 0%	
	7 +	3%	2, 5%	1, 2%	2, 3%	0, 0%	3, 5%	
**Allied Health Professional**	0	75%	66%	79%	72%	77%	81%	0.418
	1	8%	5%	2%	15%	9%	6%	
	2–3	10%	17%	7%	11%	11%	6%	
	4–6	6%	12%	11%	3%	2%	5%	
	7 +	1%	0%	2%	0%	2%	2%	

^a^*P*-value from from Kruskal–Wallis test

### Factors influencing decision to attend ED

#### Referral from community services

Prior to ED attendance 64% of participants accessed some form of community services (GP, Walk-in Clinic, PHN, AHP, Occupational Health, Pharmacist, Optician) with significant differences observed across ED sites (MRHT 74% vs. SJUH 52%; *p* < 0.05). GP services were accessed most frequently (60%) by all participants ranging from 48% in SJUH to 71% in MRHT (*p* = 0.07).

Participants were asked about their decision regarding GP consultation prior to ED attendance (Table 3). The most frequent response to this question was; “Yes, I saw my GP and was told to go to the ED” (38%). Responses varied across sites ranging from 22% in SJUH to 57% in UHK (*p* < 0.001). Teleconsultations with GPs, followed by ED referral were also relatively common across all sites (19%). Participants who did not consult a GP frequently reported that “My problem is best dealt with in the ED” (17%). This was reported least frequently by patients in UHK (10%) and most frequently (25%) by patients in SJUH. In line with this, and also in terms of accessibility of services 6% of patients in SJUH reported that they did not attend a regular GP.

**Table 3:** General practitioner consultation prior to attendance at the ED (*n* = 306)

**Did you consult your GP prior to ED Attendance?**^a^	**TOTAL**
Yes, I saw my GP and was told to go to the ED	38%
Yes, I had a phone consultation with my GP and was sent to ED	19%
Yes, I tried but could not contact my GP	3%
Yes, I saw my GP but was unhappy with the treatment	< 1%
No, I thought that my problem is best dealt with in the ED	17%
No, the GP surgery was closed	4%
No, I do not have a GP	2%
No, I thought my GP would refer me to the ED	2%
No, I did not want to bother my GP	< 1%
No, I am not happy with my current GP	< 1%
No, the GP is further away than the ED	0%
No, some other reason^b^	22%

^a^This question in the survey allowed for multiple responses

^b^Referral from other service (e.g. Occupational Health), Immediate care/Ambulance required etc.)

#### Health service awareness – alternative care pathways for emergencies

Participants were asked about awareness of alternative pathways for emergency care, defined specifically by the ability to name local services including Injury Units and OOH-GP services. Injury Units are not in operation in the Midlands or in the South West and therefore MRHT and UHK were excluded from this analysis. Awareness of Injury Units was 35% in patients at UHL compared to 9% in SVUH and 2% in SJUH (*p* ≤ 0.0001). Awareness of OOH-GP services in the full cohort was 58% and was highest in MRHT at 90% and lowest in SVUH at 40% (*p* ≤ 0.0001).

#### Reasons for ED attendance

In terms of general reasons for attending the ED, most participants (70%) believed it was the “best place” for emergency care (Table 4). This response was consistently high among all participants but did also vary across sites ranging from 64% in SJUH to 79% in UHL (*p* = 0.09). Another common reason for attendance in this study was due to lack of awareness of other services (30%) which ranging from 20% (UHK) to 41% (SVUH) or of the availability of other services open at the time (7%).

**Table 4:** Reasons for attendance at the ED in BDBP participants (*n* = 306)

**General Reasons for ED Attendance**	**TOTAL**
The ED is the best place for my problem	70%
I’m unaware of other services to treat me for this problem	30%
My family told me to come to the ED	12%
I don’t know what other services are open at this time	7%
It is easy for me to get to the ED	6%
I attended the ED before and I was happy with it	6%
I think I will be seen quicker here than at any other service	6%
I usually come to ED with a medical problem	5%
I could not afford to go anywhere else	2%
**Specific Reasons for ED Attendance**	**TOTAL**
I consider this condition to be an emergency	48%
I thought I needed an x-ray or scan	31%
I thought I might need to go into hospital	29%
I need reassurance that my illness/injury is not serious	23%
I wanted to see a doctor or a nurse as soon as possible	20%
I came to the ED to get a second opinion	11%
I thought I might need a blood test	9%
I wanted to see a specialist	7%
I thought I needed the wound treated	4%
I thought I might need a tetanus injection	3%
I am on a waiting list and I thought this would speed it up	1%
Other Reason E.g. GP Referral	23%

^a^These questions in the survey allowed for multiple responses

^b^Similar questionnaire responses were combined for the purposes of analysis (e.g. suspected fracture/X-ray required)

With regard to specific reasons for attendance, most patients considered their condition to be an emergency (48%). This response was common among participants but again significant differences were observed across hospital sites (MHRT 27% vs. SJUH 67%; *p* ≤ 0.001). Participants frequently thought hospital admission may be necessary (29%) which ranged from 10% in MRHT to 40% in SVUH. Additional reasons for ED attendance included; to receive an x-ray or scan (31%), a need for reassurance (23%), to see a nurse or doctor quickly (20%), to see a specialist (UHK 0% vs. UHL 18%; *p* ≤ 0.001) or to obtain a second opinion (SJUH 3% vs. UHL 25%; *p* ≤ 0.001).

### Clinical outcomes

The most common clinical presentation to the ED was Musculoskeletal (e.g. Back pain) 24%, followed by Cardiovascular (e.g. Chest pain) at 16% with Gastrointestinal (e.g. vomiting) and Trauma (e.g. stabbing) at 13% (Table 5). Significant differences were observed across sites with regard to Presenting Complaint (*p* ≤ 0.01). Over a third of participants (38%) reported a duration of presenting complaint of < 1 day while another 38% had symptoms for > 7 days prior to presentation. Worry levels varied significantly across sites (Median 7; IQR 5–8; *p* ≤ 0.01) while no statistical difference in pain levels was observed (Median 6; IQR 3–8; *p* = 0.386).

**Table 5:** Clinical characteristics of study participants (*n* = 306)

Category	Variable	TOTAL (*n* = 306)	MRHT (Pre-pandemic) *n* = 41	UHL (Containment) *n* = 57	SVUH (Level 2) *n* = 77	SJUH (Level 5) *n* = 67	UHK (Level 3) *n* = 64	*P* value
**Presenting Complaint**^a^	Musculoskeletal	24%	24%	19%	22%	24%	28%	0.01
	Cardiovascular	16%	12%	21%	20%	13%	11%	
	Gastroenterological	13%	2%	19%	17%	9%	14%	
	Trauma	13%	22%	5%	9%	19%	11%	
**Duration of Complaint**	< 1 day	38%	42%	28%	39%	37%	42%	0.674
	1–2 days	9%	12%	12%	8%	8%	8%	
	3–7 days	15%	10%	21%	17%	9%	17%	
	> 7 days	38%	37%	39%	36%	46%	33%	
**Worry Scale 1–10**	Median	7	7	7	7	8	7	0.01
	IQR	5–8	4–8	5–8	5–8	7–10	5–8	
**Pain Scale 1–10**	Median	6	6	6	5	7	6	0.386
	IQR	3–8	4–9	3–8	2–8	4–8	3–7	
**Triage Category**	Very Urgent	20%	10%	28%	30%	25%	0%	0.001
	Urgent	55%	49%	61%	44%	49%	74%	
	Standard	23%	37%	9%	25%	25%	25%	
	Non-Urgent	2%	5%	2%	1%	0%	2%	
**Length of Stay (LOS)**	Median (h)	5.4	3.5	7.3	4.5	5.6	5.2	0.01
	IQR	2.6–8.6	2.3–7.1	4.2–11.2	2.5–6.9	3.2–8.2	1.9–10.1	
	Range	0.0–67.1	0.5–12.5	0.3–67.1	0.0–14.4	1.1–19.2	0.2–19.4	
	< 1 h	6%	2%	4%	12%	0%	10%	
	1-2 h	9%	15%	4%	7%	5%	14%	
	2-4 h	23%	34%	14%	23%	26%	22%	
	4-8 h	33%	27%	33%	43%	42%	19%	
	8-16 h	25%	20%	37%	16%	23%	29%	
	16-24 h	4%	2%	7%	0%	4%	6%	
	> 24 h	< 1%	0%	2%	0%	0%	0%	
**Disposition**	Admitted	32%	26%	33%	29%	36%	37%	0.591
	Discharged	65%	71%	60%	70%	65%	61%	
	Did Not Wait	2%	3%	5%	1%	0%	2%	
	Transferred	< 1%	0%	2%	0%	0%	0%	

^a^Presenting Complaint was characterised as per the categories outlined in the RCEM Syllabus in the UK, which is aligned with the Irish Association of Emergency Medicine (IAEM) Training Standards in Ireland

^b^Interquartile Range (IQR) 25^th^- 75^th^ percentile. *P*-value from Chi^2^-tests for categorical variables and from Kruskal–Wallis test for continuous variables

The triage category most frequently recorded in this study was for “Urgent” cases at 55%, and “Non-urgent” cases were least frequently recorded at 2%. Significant differences were observed between sites by triage category (*p* ≤ 0.001). “Very Urgent” cases were most frequently recorded in SVUH (30%) whereas there were no cases triaged as “Very Urgent” in UHK during the study. Median LOS in the ED for all participants was 5.4 h (IQR 2.6–8.6) and ranged from 3.5 h in MHRT to 7.3 h in UHL (*p* ≤ 0.01). By LOS category, across all sites, most participants stayed in the ED for 4–8 h (33%). In the full BDBP cohort 58% of patients waited > 4 h in the ED which ranged from 49% of patients in MRHT to 79% of patients in UHL. Comparisons across age cohorts indicated that older adults aged > 65y had significantly longer lengths of stay in the ED than younger adults aged 18-39y (Median stay 7.1 h vs. 4.0 h; *p* < 0.01).

In terms of disposition outcome most participants were discharged home following treatment in the ED (65%). The lowest rate of hospital admissions was recorded in MHRT at 26% and the highest rate was recorded in SJUH at 37% however, there was no significant difference across sites in terms of disposition outcome (*p* = 0.591).

## Discussion

In this national study of ED attendances in Ireland, significant differences were observed in the demographic and clinical characteristics of patients across regional hospital sites which will have implications for planning of future services and the implementation of the Sláintecare Strategy.

The demographic profile of patients differed nationally across ED sites in terms of age, socioeconomic status, healthcare coverage and proximity of health services. These findings are in direct agreement with previous Irish research which reported that at a population level, older demographics, socioeconomic deprivation and rurality were major sources of geographical variation in emergency admissions [9]. More recent Irish data suggests that up to 60% of older adults presenting to the ED are admitted for inpatient care [30] and a recent systematic review found that increased presentations by older patients with complex, chronic conditions is a significant driver of ED crowding [19]. This is supported by the data in this study with adults aged > 65y having significantly longer lengths of stay in the ED (7.1 h vs. 4.0 h; *p* < 0.01) than adults aged 18-39y, reflecting the more complex care needs of older adults which results in prolonged treatment times in the ED. Internationally Morley et al. reported similar findings with regard to regional variations in ED utilisation and increased presentations in older adults across all triage categories [31]. The differences in age profiles across ED sites in the BDBP study should be used to inform the evidence-based planning of future emergency care for older adults. To date, the roll-out of these services has been ad hoc with “Frailty at the Front Door” in place at some EDs and prehospital frailty response teams also operating in limited areas (e.g. Dublin city). However, in this study the oldest patient cohort was associated with a rural hospital (MRHT) which further demonstrates the need for evidence-based service planning.

A total of 9% of patients in the BDBP study live ≥ 50 km from their nearest ED. Residing an extremely long distance from definitive care can compromise patient safety and a Norwegian study found that increased distance is associated with lower utilisation of out-of-hours services, even in the most acute cases [32]. This illustrates the importance of adequately resourcing the National Ambulance Service and particularly services such as Helicopter Emergency Medical Services (HEMS; Medevac 112) to ensure equity of access to healthcare for patients living in remote areas, particularly for time-sensitive conditions such as cardiovascular emergencies and multi-system trauma [33].

With regard to the social determinants of health, significant differences in socioeconomic status were observed across sites (*p* ≤ 0.001), and access to care appeared to be impacted most in inner city Dublin and also in isolated rural communities. It has previously been reported in the UK that avoidable ED attendances are mostly driven by underlying deprivation [34]. Similarly, Unwin et al. recently reported that patients presenting to an Australian ED with non-urgent conditions were almost four times more likely to live in the most socioeconomically deprived communities [35]. In the BDBP Study the hospital sites with the highest numbers of patients living in disadvantaged areas (UHL 21% and SJUH 22%) also recorded the highest median LOS figures (UHL 7.3 h and SJUH 5.6 h) which raises the question of equitable service availability and accessibility. It also highlights the fact that expansion of ACPS and delivery of the “Right Care, Right Time, Right Place” must factor in these regional differences in order to adequately address the needs of local populations.

The regional socio-economic differences observed here require Public Health interventions at an upstream point. Health improvements at societal level have the potential for amplification downstream in regard to policies which will directly impact on ED crowding. The relatively high levels of health services utilisation in this study suggests the presence of underlying conditions and comorbidities in this population which is driving their health-seeking behaviour and which may also need addressing from a Public Health perspective. Comorbidities were not specifically addressed in this study however it is an important area requiring further research in the future. The burden of chronic diseases such as cancer, cardiovascular disease, diabetes, and respiratory diseases could be reduced through evidence-based strategies focused on behavioural risk factors, including physical inactivity, diet-related factors and obesity, and the use of addictive substances such as tobacco, alcohol and illicit drugs [35].

In terms of patient awareness of alternative care pathways (ACP) to the ED this research found that awareness of OOH-GP was relatively high at 58% (range 40–90%) however, even in areas with existing Injury Units the awareness of this service was much lower. Nationally there are regional variations with regard to access to OOH-GP, Injury Units and other types of ACP (e.g. Frailty Teams) which likely also impact on ED utilisation. Musculoskeletal injuries were the most common reason for presentation to the ED in this study (24%) and almost a third of patients (31%) reported presenting to the ED for an x-ray or scan. This suggests that improved awareness of, and expansion of ACP could potentially decrease ED attendance, which would be particularly important in the context of reducing ED crowding during the COVID-19 pandemic. In support of this theory, awareness of Injury Units was highest in participants from UHL, which consistently suffers the highest levels of overcrowding in the country, as reported by the Trolley Watch statistics compiled by the Irish Nurses and Midwifes Organisation (INMO) [36]. Also of note in these findings is the fact that 38% of patients reported symptoms for > 7 days prior to ED presentation suggesting a non-emergent condition which may potentially be an avoidable ED attendance which could be treated in primary care. Adequate resourcing of primary care is essential to reduce the burden on EDs. Recent Irish data indicates that GP supply is an issue nationally, with an increased density of GPs required in rural and deprived areas [7] However, access to primary health care alone does not fully explain the differences in potentially avoidable ED attendance patterns. This research found that 64% of patients first engaged with health services in the community, with most patients (60%) initially accessing a GP with subsequent referral to the ED. It should be acknowledged that this figure may be higher in this study than previously reported due to COVID-19 related factors and fear of infection at the ED. Conflicting evidence exists in the research literature as to whether increased access to primary care can reduce ED utilisation [34, 37], which may relate to local factors including GP capacity and options for ACP. [34, 37]. However, it is clear that additional diagnostics facilities are required outside hospitals and acknowledging this, the Irish College of General Practitioners recommended immediate expansion of radiological, cardiac and endoscopic investigations for all patients in 2016 [38].

Additionally, ACP could be developed through expansion of the roles of other AHP within PCTs including Physiotherapists, Occupational Therapists and Pharmacists. Paramedics are also experienced in providing acute care, palliative and end-of-life care, which can complement the current care being provided by PCTs. A recent systematic review found that both paramedics and nurses, with additional training and an enhanced skillset, can significantly reduce ED presentations and hospital admissions [39]. In Ireland, the Advanced Nurse Practitioner [40] and Community Care Paramedic [41] are relatively new roles with an extended scope of practice which have potential for the development of other ACP with PCTs.

Past experience in the ED, affordability, ease of access and timeliness of care did not appear to be significant drivers of ED presentation in this study, which is in agreement with the findings of Kraaijvanger et al. 2016 [42] but also conflicts with some previous international studies [43, 44]. This again highlights the issue of ED crowding as being one of the most complex challenges in the field of health services research and public health. In terms of other common reasons for ED attendance, ED patients frequently reported that their condition was an emergency and that the ED was the “best place for treatment, which does align well with the international literature [45, 46]. Reasons for patient attendance differed across sites again suggesting differing care needs in the populations served by these regional EDs.

In terms of clinical outcomes, significant differences were observed across sites by triage category (*p* ≤ 0.001) and for LOS in the ED (*p* ≤ 0.01). Regional differences in operational metrics such as LOS relate to local policies with regard to patient disposition and bed capacity at each hospital site, which contributes to access block in the ED. According to the Trolley Watch statistics, crowding was recorded at all ED sites during the BDBP study with the number of patients on trolleys ranging from n = 2 in MRHT to *n* = 63 IN UHL [36]. In line with this median LOS was lowest in MRHT (3.47 h) and highest in UHL (7.25 h) during the BDBP Study. Access block and ED crowding are not solely ED issues and sustainable system-wide solutions are required to address capacity and flow in the wider hospital and within community services.

### Limitations

This research paints a picture of lower acuity attendances to regional Irish EDs over the course of a year, therefore findings may not be entirely generalisable to other settings. Data collection was delayed by the COVID-19 pandemic and this was not statistically factored into site comparisons, which is a confounding variable in the study. Presentations related to Mental Health or for drug/alcohol intoxication were excluded from this study, due to ethical issues around obtaining consent to interview a distressed patient. However, it should be acknowledged that these populations may have been disproportionally affected by the COVID-19 pandemic during 2020. The questionnaire utilised in the study was designed for the purposes of this research and has not been externally validated. Proximity to health services (GP and ED) was self-reported by patients and while it is acknowledged that this is a crude measure, it was considered sufficient for the purposes of this study. Strengths of this study include the level of detail captured in relation to patterns of healthcare utilisation and health seeking behaviour.

### Implications

ED crowding is a significant issue in health services delivery that poses a risk to global health, which has only been exacerbated since the onset of COVID-19. Most Irish EDs have inadequate infrastructure to enable physical distancing and isolation capacity is limited meaning that a crowded ED environment will increase transmission rates of infectious diseases.

Solutions to ED crowding must be data-driven and evidence-based in order to provide meaningful information for policymakers in the health services, and this is particularly important in the context of future pandemic preparedness. In order to provide “Better Data for Better Planning”, the BDBP study has identified regional and socioeconomic differences in the drivers and patterns of ED attendance and highlighted important factors that influence ED presentation from the patient perspective.

## Conclusions

The implementation of new strategies for integration of acute care in the community must acknowledge and plan for key issues that contribute to ED crowding as a universal approach is unlikely to be implemented successfully due to regional factors. The “Trolley Crisis” and practising of “Corridor Medicine” in our hospitals should be consigned to the pre-pandemic era. Expanding access to primary care and sustainable integration of health services could reduce avoidable ED attendances and direct patients to the “Right Care, at the Right Time, in the Right Place, by the Right Team.

## Supplementary Information

**Additional file 1:** **Supplementary Table S1.** Service Utilisation of Health and Social Care Professionals in the BDBP Study.

## Data Availability

The datasets analysed during the current study are available from the corresponding author on reasonable request.

## Abbreviations

BDBP: Better Data, Better Planning
ED: Emergency Department
GP: General Practitioner
HSE: Health Service Executive
UHK: University Hospital Kerry
MRHT: Midlands Regional Hospital Tullamore
OOH: Out-of-hours
OPD: Out-Patient Department
UHL: University Hospital Limerick
RN: Research Nurse
SVUH: St. Vincent’s’ Hospital Dublin
SJUH: St. James University Hospital Dublin
