# Supplementary material for: The “better data, better planning” census: a cross-sectional, multi-centre study investigating the factors influencing patient attendance at the emergency department in Ireland
Source: BMC Health Serv Res. 2022 Apr 9;22:471. doi: 10.1186/s12913-022-07841-6 (PMC8994521; doi:10.1186/s12913-022-07841-6)
Supplement: Supplementary file 1 — Additional file 1: Supplementary Table S1. Service Utilisation of Health and Social Care Professionals in the BDBP Study. [file 12913_2022_7841_MOESM1_ESM.docx]

**Additional File 1 – Supplementary Table S1**

Table S1 Service Utilisation of Health and Social Care Professionals in the BDBP Study

| **Utilisation of Other Services and Additional Details*** | | | | | | | | |
| --- | --- | --- | --- | --- | --- | --- | --- | --- |
| **Health Service** | **Frequency** | **TOTAL**  **(n=306)** | **MRHT**  **(n=41)** | **UHL**  **(n=57)** | **SVUH**  **(n=77)** | **SJUH**  **(n=67)** | **UHK**  **(n=64)** | **P value** |
| Hospital Day Case | ≥1 | 18% | 24% | 12% | 17% | 19% | 17% | 0.628 |
| Day Hospital Consult | ≥1 | 7% | 2% | 4% | 7% | 13% | 5% | 0.109 |
| Physiotherapy | ≥1 | 15% | 27% | 14% | 16% | 13% | 11% | 0.250 |
| Occupational Therapy | ≥1 | 2% | 5% | 2% | 0% | 3% | 8% | 0.420 |
| Speech & Language | ≥1 | <1% | 0% | 2% | 0% | 0% | 0% | 0.357 |
| Psychology/Counselling | ≥1 | 3% | 0% | 0% | 4% | 6% | 2% | 0.173 |
| Respite Care | ≥1 | 1% | 0% | 2% | 0% | 0% | 3% | 0.266 |
| Pharmacy | ≥1 | 16% | 29% | 7% | 20% | 13% | 13% | 0.032 |

^a^P-value from Chi^2^-test
